# Editorial: LncRNAs in Cancer Metastasis and Therapy Resistance

**DOI:** 10.3389/fonc.2021.813274

**Published:** 2021-12-10

**Authors:** Aamir Ahmad, Palmiro Poltronieri, Shahab Uddin

**Affiliations:** ^1^ Anesthesiology and Perioperative Medicine, University of Alabama, Birmingham, AL, United States; ^2^ Institute of Sciences of Food Production, Italian National Research Council, Lecce, Italy; ^3^ Academic Health System, Hamad Medical Corporation, Doha, Qatar

**Keywords:** competing endogenous RNAs, MicroRNAs, structured RNAs, cancer biology, metastasis, resistance

Long noncoding RNA transcripts (lncRNAs) are being revealed by complementary DNA cloning and genome tiling array studies in animals. Differently from structured RNAs (RNAs, snoRNAs, etc), lncRNAs have been named regulatory RNAs (1, 2). With the finding that microRNAs can be sponged by lncRNAs, named competitive endogenous RNAs (ceRNAs), an alternative function of these RNA molecules is in the assembling of protein complexes, thanks to a tridimensional RNA structure able to interact with proteins involved in transcription, mRNA splicing, epigenetic regulation, chromatin opening or compaction (3). It was shown in bacteria that any species of RNA with a sequence complementary to an mRNA may regulate its stability (avoiding access to ribonucleases) or may block translation by masking the Shine-Dalgarno region or giving access to an alternative internal transcription start site.

LncRNAs are relatively long RNA sequences (more than 200 nucleotides) long that impact the cellular gene expressions and functions in ways that are just beginning to be explored. The metastasis of human cancers as well as acquired resistance against the administered therapeutics are two major factors responsible for the cancer-associated mortality. In a small number of cases lncRNAs are translated into short peptides, and in this case both RNAs and peptides may have a functional role. The big question is whether these transcripts are relevant. To this aim, there are today technologies available such as transient RNA silencing, gene overexpression, and high-throughput CRISPR/Cas genome editing methods that in principle could generate cell lines with all the possible combinations of silenced genes.

In search of novel biomarkers as well as potential therapeutic targets of cancer metastasis and cancer drug resistance, the focus in recent years has turned to lncRNAs. What started with studies on relative expression of lncRNAs with the aim to define their utility as diagnostic biomarkers, has evolved into explorations of the possible functional role that these ncRNAs possibly play. One of the relatively more explored functions of lncRNAs is their ability to sponge and regulate the expression of microRNAs (miRNAs), the other ncRNAs that are much shorter in length. This indirectly opens the flood gates to endless possibilities of regulation of gene expression, as miRNAs represent a much better explored subtype of ncRNA. However, regulation through miRNAs is perhaps not the only mode of lncRNAs functionality, as being revealed through continued interest and evaluations.

The list of lncRNAs known to cancer researchers is ever expanding, as is the evidence linking their possible role as diagnostic as well as prognostic markers. Concerning lncRNAs, there are published reports on lncRNAs in almost all cancers that affect humans. There is evidence supporting the role of specific lncRNAs in metastasis in general as well as organ-specific metastasis of different cases. Also, lncRNAs seem to be involved in acquired resistance against a majority of chemical therapeutics as well as radiotherapy. This Research Topic collection includes research and review articles focusing on lncRNAs in cancer types, in metastasis and therapy resistance models. The 38 papers in this Research Topic describe lncRNAs deepening on mechanistic insights using *in vitro* or *in vivo* models and RNA silencing techniques.

We thank the editorial team at Frontiers for their support in the process of reviewing and publishing this Research Topic, “*lncRNAs in Cancer Metastasis and Therapy Resistance*”, and wish that new research work will get insight from this collection to continue to produce data and information on the large set of human lncRNAs in cell physiology and in the pathology of cancers.

## Author Contributions

PP and AA wrote the manuscript. SU reviewed it. All authors contributed to the article and approved the submitted version.

## Conflict of Interest

The authors declare that the research was conducted in the absence of any commercial or financial relationships that could be construed as a potential conflict of interest.

## Publisher’s Note

All claims expressed in this article are solely those of the authors and do not necessarily represent those of their affiliated organizations, or those of the publisher, the editors and the reviewers. Any product that may be evaluated in this article, or claim that may be made by its manufacturer, is not guaranteed or endorsed by the publisher.
